# Feasibility of Implementation and the Impact of a Digital Prehabilitation Service in Patients Undergoing Treatment for Oesophago-Gastric Cancer

**DOI:** 10.3390/curroncol30020128

**Published:** 2023-01-30

**Authors:** Krishna Moorthy, Laura J. Halliday, Nigel Noor, Christopher J Peters, Venetia Wynter-Blyth, Catherine E Urch

**Affiliations:** 1 Department of Surgery and Cancer, Imperial College, London W2 1NY, UK; 2 Imperial College Healthcare NHS Trust, London W2 1NY, UK; 3 Onkohealth Ltd., Edgware HA8 7EB, UK

**Keywords:** oesophageal cancer, prehabilitation, digital health, surgery, preoperative, postoperative, complications

## Abstract

Background: Home-based and supervised prehabilitation programmes are shown to have a positive impact on outcomes in patients with oesophago-gastric (OG) cancer. The primary aim of this study was to establish the feasibility of delivering a digital prehabilitation service. Methods: Patients undergoing treatment for OG cancer with curative intent were recruited into the study. During the COVID-19 pandemic, patients were offered a digital prehabilitation service. Following the lifting of COVID-19 restrictions, patients were also offered both a hybrid clinic-based in-person service and a digital service. Implementation and clinical metrics from the two prehabilitation models were compared. Results: 31 of 41 patients accepted the digital service (75%). Of the people who started the digital programme, 3 dropped out (10%). Compliance with the weekly touchpoints was 86%, and the median length of programme was 12 weeks. Twenty-six patients enrolled in the in-person service. Two patients dropped out (10%). Average compliance to weekly touchpoints was 71%, and the median length of programme was 10 weeks. In the digital group, sit to stand (STS) increased from 14.5 (IQR 10.5–15.5) to 16 (IQR 16–22); *p* = 0.02. Median heart rate recovery (HRR) increased from 10.5 (IQR 7.5–14) to 15.5 (IQR 11–20) bpm; *p* = 0.24. There was a significant drop in distress (median 3 (IQR 0–5) to 1 (IQR 0–2); *p* = 0.04) and a small drop in anxiety (median 3 (0–5) to 2 (0–3); *p* = 0.22). There was no difference in the postoperative complication rate and length of hospital stay between the two groups. Discussion: This study has shown that digital prehabilitation can be delivered effectively to patients with OG cancer, with high engagement and retention rates. We observed improvements in some physical and psychological parameters with the digital service, with comparable clinical outcomes to the in-person service.

## 1. Introduction

Around 17,000 patients are diagnosed with oesophago-gastric cancer in the UK each year, of which a third undergo radical treatment. This includes neoadjuvant chemotherapy (NAC), with or without radiotherapy, followed by a major surgical procedure. In total, 60% of patients have at least one complication, and the median length of stay is around 11 days [1,2]. The reasons for these outcomes are multifactorial; patients are often elderly, physically deconditioned, and the use of NAC leads to further reduction in cardio-respiratory fitness. High postoperative morbidity can result in a delayed recovery, long-term disability, and poor survival [3,4,5]. 

Prehabilitation, a relatively new concept, is the process of using the time prior to surgery to improve the functional capacity of an individual so they can better withstand the stress of surgery [6]. There is increasing evidence of the effectiveness of prehabilitation in improving preoperative fitness and reducing postoperative complications in many surgical specialties, including OG cancer surgery. We have previously demonstrated the impact of a hybrid clinic–home-based model of prehabilitation on health and clinical outcomes in patients undergoing curative treatment for oesophago-gastric cancer [7,8].

Due to the challenges of delivering the programme during the COVID-19 pandemic, we sustained the service through a digital prehabilitation model. Through this initiative, it was also anticipated that we could provide access to prehabilitation to patients across our partner cancer network, as prior to this programme patients from the network did not receive prehabilitation. 

This study aims to firstly explore the feasibility of implementing a digital prehabilitation service in cancer patients, secondly to measure the impact of digital prehabilitation on physical fitness and psychological wellbeing, and finally to compare the impact of digital prehabilitation on postoperative outcomes against in-person clinical service. 

## 2. Materials and Methods

### 2.1. Patient Recruitment

Patients undergoing treatment for OG cancer with curative intent between September 2020 and September 2021 were recruited into the study. Referrals were received from North-West London Network (6 hospital sites) and the Bedfordshire and Hertfordshire network (2 site). 

In the period from Sept 2020 to Feb 2021, patients were only offered the digital service due to staffing constraints in the in-person service as a consequence of the COVID-19 pandemic. The in-person service resumed in February 2021 with the lifting of the COVID-19 lockdown restrictions. From then on, patients were first offered the digital arm. If they were unwilling or unable to access the digital service, they were referred to the in-person service (hospital physiotherapist). However, clinical teams still had the option of referring patients to the hospital physiotherapist directly, as she regularly attended outpatient clinics. 

### 2.2. Study Interventions

#### 2.2.1. Digital Prehabilitation Service 

The digital prehabilitation service (DPS) is a behaviour change health coaching programme for people undergoing and/or preparing for cancer treatment. The features available in the app during the study period consisted of a screening questionnaire (Cancer Health Checker), video platform for consultations, messaging, and wearable device connectivity. All patients undergoing the digital service were offered a Fitbit Inspire 2 device (Fitbit, San Francisco, NC, USA). The digital service was offered through an app and a non-app pathway. App-pathway patients downloaded the app (Onkohealth Ltd., London, UK) from the Apple and Google app stores. Non-app-pathway patients received the digital service through e-mail, a web browser for surveys, web-based video conferencing, and the wearable technology manufacturer’s app.

The digital service was delivered by a team of behaviour change coaches with a background in dietetics, physiotherapy, and nursing. Patients were allocated a coach who covered all aspects of prehabilitation (exercise, dietetics, and psychological wellbeing). Any specialist services, such as specialist dietetic support and clinical psychologist, were provided through the hospital services. This model is aligned to Macmillan’s tiered framework for prehabilitation delivery [9].

The exercise aspect of the digital service is similar to in-person service [8], but the assessment and training is virtual (via video consultation). Exercise plans are intended to optimise whole-body aerobic fitness and muscle strength and are personalised to each individual. Only behaviour change aspects of diet such as quality and eating behaviours are modified. As all patients in this study were diagnosed with oesophago-gastric cancer, they were all reviewed by the local hospital specialist dietitians (in line with NICE guidance). Psychological wellbeing advice consists of addressing and optimising emotional wellbeing and self-efficacy. Patients with high levels of psychological morbidity were referred to a clinical psychologist.

#### 2.2.2. In-Person Prehabilitation Service

The details of the in-person prehabilitation model have previously been published [8]. In brief, a physiotherapist undertakes a physical health assessment including cardio-respiratory fitness, muscle strength, and balance. A personalised whole-body exercise programme is agreed on with the patients. The only difference to our previously published programme, in this study, was that only the weekly touchpoints could be conducted through video consultations if needed, instead of a telephone.

### 2.3. Study Outcomes

Engagement with digital prehabilitation was estimated by calculating the proportion of people for whom the feature was available to those who engaged with the feature. The different features measured in this study were the screening questionnaire (Cancer Health Checker) and wearable device connectivity. This was defined as data being available for >3 days of the week for >50% of the time in the programme. 

Feasibility measures included attendance at the baseline (T1) and postprogramme (T2) physical performance assessment sessions (as below); compliance, which was defined as a ratio of the weekly contact/touchpoints attended per week to the total weeks in the programme; and retention/dropout rate, defined as the proportion of patients who completed the programme. 

In the digital programme, patients self-reported physical activity levels during the programme using a modification of the International Physical Activity Questionnaire (IPAQ). Patients were asked to quantify the time spent per week in moderate and vigorous physical activity. METs mins per week were calculated as the product of time spent multiplied by 3.5 METs for moderate intensity activity and 6 METs for vigorous intensity activity [10]. Both moderate and vigorous intensity activities were added to derive a total physical activity score per week (METs mins per week) [11]. Average step count per week was also recorded. 

A virtual physical performance assessment was undertaken at the beginning and completion of the neoadjuvant treatment period (T1 and T2), which consisted of a short physical assessment battery. This is a modified version of the Short Physical Performance Battery [12] for remote assessment validated for people with cancer. This included:

- Cardio-respiratory fitness assessed by performing the 1 min sit-to-stand test and measuring the heart rate at rest and at the end of the test and 1 min after recovery [13]. Heart rate recovery (HRR) was end of exercise heart rate (beats per minute) minus the heart rate at 1 min recovery. Higher values are associated with improvements following exercise-based cardiac rehabilitation programmes [14].
- Lower limb muscle strength assessed using the 30 s ‘sit-to-stand test’ [15].

Psychological wellbeing was assessed by self-report of Anxiety and Distress using the Emotional Distress Scale [16]. 

Within the in-person programme, physical fitness was assessed using the Chester Step Test to calculate predicted VO2max. Details of this assessment have previously been published (Halliday). Lower limb muscle strength was also assessed using the 30 s ‘sit-to-stand test’ [15].

Postoperative outcomes were recorded for both the digital and in-person groups. Complications were defined according to the Esophagectomy Complication Consensus Group (ECCG) guidelines [17] and graded using the Clavien Dindo classification. Postoperative pneumonia was defined as previously published [8]. Length of hospital stay was also recorded. All patients received standardised postoperative care based on the surgical team’s enhanced recovery protocols (ERP).

### 2.4. Consent and Regulatory Approvals

All patients in the digital programme gave informed consent to sharing their data as per GDPR regulations. Ethical approval for the retrospective analysis of data for publication purposes was granted by the UK Health Research Authority (Ref 268837).

### 2.5. Statistical Analysis

Changes in health outcomes (physical and psychological) during prehabilitation were assessed using the Wilcoxon signed-ranks test and the chi-squared test where appropriate. 

Baseline characteristics and postoperative outcomes were compared between digital and in-person using chi-squared tests for categorical outcomes. For continuous outcomes, either Mann–Whitney or unpaired *T* tests were used, depending on distribution. Two-tailed tests were used throughout, with a significance level of *p* < 0.05. Statistical analysis was performed using SPSS version 25 (IBM, New York, NY, USA). 

## 3. Results

### 3.1. Recruitment and Retention

**Figure 1:**
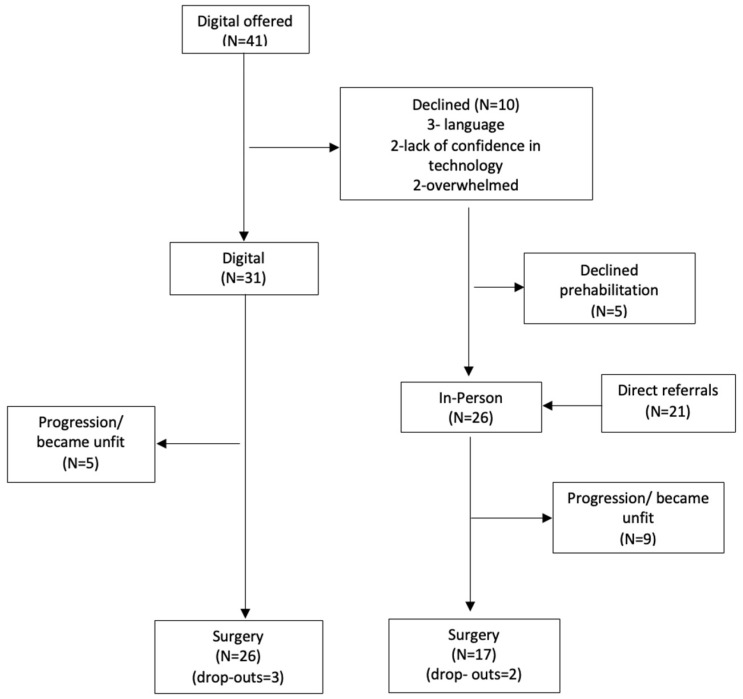
Flow chart of patients recruited.

Recruitment and retention to the digital programme is shown in Figure 1. A total of 41 patients were offered the digital service, of which 31 agreed to participate (75%).

Of the 10 patients who declined, 5 declined prehabilitation completely and 5 started the in-person service. Reasons for declining the digital programme included language problems (*n* = 3), lack of confidence in technology (*n* = 2), and feeling overwhelmed (*n* = 2). No reason was given by the other three patients.

Of the people who started the digital programme, three patients dropped out (10%) a median of 4 weeks after starting the programme. Compliance to the weekly touchpoints was 86%. In total, 100% of patients attended the T1 assessment and 55% attended the T2 session. The median time in the programme was 12 weeks.

Twenty-six patients started the in-person service. Twenty-one patients were direct referrals to the service. Two patients dropped out of the programme (10%) after a median of 4.5 weeks from starting the programme. Both patients had also declined the digital service. Average compliance to weekly touchpoints was 71%. Attendances at the T1 and T2 assessments were 46% and 15%, respectively. Median time in the programme was 10 weeks.

### 3.2. Engagement with Digital Prehabilitation

The Cancer Health Checker was available to all patients and was completed by all 31 patients (100%). The connectivity of wearables was available to all patients, but reliable data were available for only 17 of the 31 patients (55%). This improved from 4/16 (25%) in the first half of the study period to 13/15 (86%) in the second half of the study period. 

### 3.3. Physical Activity, Fitness, and Psychological Wellbeing 

Health improvement data could only be analysed in the digital arm due to low rates of completion of the T2 assessment in the in-person arm (14%). 

Table 1 shows the changes in the digital group: there was an improvement in their physical activity assessment measures. The 30 s STS increased from 14.5 (IQR 10.5–15.5) to 16 (IQR 16–22); *p* = 0.02). There was also a trend for an increase in HRR during the programme: median HRR increased from 10.5 (IQR 7.5–14) to 15.5 (IQR 11–20) bpm; *p* = 0.24. 

There were no changes in the mean self-reported physical activity (346.5 (sd = 362) METs min per week to 407 (sd = 400); *p* = 0.64) or the average step count per day (5179 (3204) to 4550 (3061); *p* = 0.55). 

There was a significant fall in Distress ((median 3 (IQR 0–5) to 1 (IQR 0–2); *p* = 0.04) and a small fall in anxiety (median 3 (0–5) to 2 (0–3); *p* = 0.22). There was no significant change in depression scores (1 (0–3) and 1 (0–2); *p* = 0.41).

### 3.4. Comparison of Postoperative Outcomes with In-Person Programme

Baseline characteristics were comparable between the two groups (Table 2), with the exception of a nonsignificant trend towards a higher median CCI score in the in-person group.

In total, 26 patients (83%) in the digital group and 17 patients (65%) in the in-person group proceeded to surgical resection. Five patients and nine patients in the respective groups had disease progression during the preoperative period, which precluded resection.

The postoperative complication rate was 50% versus 64%, the postoperative pneumonia rate was 23% versus 41%, and the postoperative hospital stays were 10.5 versus 17 days for the digital and in-person arms, respectively. There was no significant difference between the two groups. 

## 4. Discussion

This pilot study demonstrated the feasibility of delivering a fully virtual cancer prehabilitation service. Digital recruitment was 75%. Of these patients, nearly 40% did not possess mobile technology or have confidence in the use of apps, and consequently a non-app pathway was implemented using a web browser. The findings are consistent with the finding that nearly 22% of patients in the NHS do not have basic digital skills [18]. The high uptake of digital prehabilitation in this study is most likely a reflection of the higher adoption of digital technology during the COVID-19 pandemic [19]. However, it probably also reflects that we made training and support an important aspect of our implementation. Early in the project, we developed a manual that was sent to patients that went through a stepwise process on how to download and use the key features of the app, including the wearable connectivity. People also had the option of receiving one-on-one training with a digital care navigator. Unfamiliarity with the English language was a reason in a third of patients who did not take up the digital programme. There is an awareness in the NHS that more needs to be done to ensure digital inclusivity for non-English-speaking patients [18] and, moving forwards, it would be relatively straight forward to translate the text on the app.

Just 5 (8%) patients chose not to do prehabilitation altogether, and a small number of patients (10%) in both arms dropped out of the programme. We believe that this reflects that with clinical endorsement and support, a high number of patients do start and complete prehabilitation programmes. The high programme completion (84%) and compliance rates (86%) in the digital arm, seen in our study, most likely reflect the element of professional support offered to patients throughout the 12-week programme. This is in comparison with self-management apps that are associated with high drop-out rates [20]. The findings of our study are also consistent with higher engagement rates with other digital behaviour change programmes that offer some level of support [21]. Even in diabetes, where the role of digital health is well-established, human support is provided in all the digital programmes that have demonstrated clinical effectiveness [22]. In cancer, there are a number of apps that are directed at symptom management and medication adherence, but those that integrate professional support are associated with higher levels of satisfaction and lower attrition rates [23] as opposed to pure self-management apps [24].

While just over half of the patients attended the end of the programme’s virtual health assessment, this was only 14% in the in-person cohort. The reasons for this are manifold but include conflicting clinic appointments and last-minute scheduling of cancer operations. One of the challenges of hospital-based behaviour change interventions is the low uptake among patients due to difficulties with travel and with having to fit in their therapy sessions around other hospital appointments [25,26]. The challenges in the in-person service cohort could also have been exacerbated by the fear of travel to a hospital environment during the pandemic [27,28]. Patients prefer home-based interventions [29], but the lack of direct supervision may hinder adherence and impact their effectiveness [30]. A number of other prehabilitation programmes switched to digital platforms during the pandemic [31]. In addition to the ability to deliver programmes completely remotely, digital platforms offer the advantage that they can be integrated with behaviour change interventions, such as wearable devices to monitor adherence. 

We showed maintenance or improvement in physical activity, physical fitness, and psychological measures. Even maintenance of physical activity would be beneficial, as it is contrary to the drop traditionally seen in patients undergoing chemotherapy [32]. The slight discrepancy between the self-reported physical activity and the step count could reflect the inherent bias with self-report or could be a result of the problems our patients faced with connecting the wearable device with the DPS early on in the study. This was addressed through iterative developments to the technology and user training. Going forwards, we believe that human factors issues need to be acknowledged and addressed in the successful implementation of digital health solutions, especially in older patients with cancer [33,34]. The discrepancy between physical activity, which was maintained, and other exercise measures such as cardio-respiratory fitness and muscle strength, which improved, could be a result of shielding during the pandemic, where patients were more adherent with their higher intensity home-based exercises rather than indulging in any outdoor activities.

While this study demonstrated improvements in the physical assessment measures, we faced a number of challenges in undertaking the assessment remotely. We either considered or implemented other tests, such as the Six Minute Walk Test (6MWT) and the Chester Step test (CST). During the co-design of our remote programmes, our patient user group, as well as clinical team, were of the view that the 6MWT and the Incremental Shuttle Walk Test would be challenging for many patients due to the lack of even a 10 m distance (ideal distance being 30 m) [35] in most people’s homes. We attempted the CST in a small sample of patients, but this was abandoned due to concerns about undertaking a virtual stepping test in a cohort of patients who are older, frail, and prone to falls. 

We also showed a maintenance of postoperative outcomes in the patients who underwent the digital programme when comparing the outcomes in this study with our previously reported findings [8]. Although the differences were not statistically significant, there was a small trend for worse outcomes in the in-person cohort than the digital cohort. This is probably a reflection of the selection bias in our study. A total of 14 of 57 (25%) patients did not go on to having surgery. This is higher than what we have seen in our centre [8] and is probably a reflection of the impact of the pandemic on cancer treatment [36]. There was a greater number of people in the in-person service who progressed or became unfit during the course of the programme while receiving neoadjuvant therapy. Eight of the nine who did not go on to have surgery were direct referrals to the in-person programme, and the median CCI score was also higher in the in-person group. This may reflect clinical teams directly referring higher-risk patients to the in-person service due to a greater trust in a service that they were more familiar with. Nearly 90% of the patients in the in-person service were direct referrals to the service, despite the protocol stating that patients should be referred to the digital service first. It has been shown that trust and confidence on the part of clinical staff is just as important as winning the trust of patients [37] for digital health solutions to be successfully implemented. 

In addition to being able to sustain the programme during the pandemic, we were also able to extend the programme to patients from our partner network who did not have access to prehabilitation due to the distance involved in travelling to our centre. Digital solutions allow us to ‘democratise’ behaviour change interventions and address the ‘postcode’ lottery that can exist in the delivery of patient support services [38]. A centralised approach to prehabilitation is possibly also more cost-effective, as each organisation does not have to recruit, train, and retain their own staff, especially for small-volume cancer services. As prehabilitation is gaining more traction, the establishment of regional prehabilitation services and the delivery through a digital platform allows organisations to scale prehabilitation in a cost-effective manner [9].

## 5. Limitations

This study is a pragmatic real-world implementation of a digital prehabilitation programme, which replicated our previous hybrid clinic–home-based programme. There are a number of limitations. We did not have a control group who did not receive prehabilitation, but we compared prehabilitation processes and outcomes with a parallel group of patients who underwent the in-person programme. The engagement with the latter was most likely impacted by the pandemic, and the results of which were likely influenced by a selection bias. There was also a difference in the median number of weeks in the programme. This was likely a result of there being more people who either dropped out of the programme or who had disease progression in the in-person cohort. We also faced a number of technology issues, especially with the interoperability of the wearable device with the DPS, which could have affected the physical activity measurement. During this study, we only implemented the features that facilitated the remote delivery of our programme. We developed further behaviour change features that were only available to the last four patients recruited in this study. Future research will explore the impact of these new features.

## Figures and Tables

**Table 1:** Changes in physical and psychological health measures.

	Pre	Post	*p* Values
30 s STS Median (IQR)	14.5 (10.5–15.5)	16 (16–22)	0.02
HRR Median (IQR)	10.5 (7.5–14)	15.5 (11–20)	0.24
Self-reported PA METs mins per week Mean (sd)	346.5 (362)	407 (400)	0.64
Step count Mean (sd)	5179 (3204)	4550 (3061)	0.55
Distress Median (IQR)	3 (0.5)	1 (0–2)	0.04
Anxiety Median (IQR)	3 (0–5)	2 (0–3)	0.22
Depression Median (IQR)	1 (0–3)	1 (0–2)	0.41

(30sSTS—30 s sit-to-stand test; HRR—heart rate recovery; IQR—interquartile range; sd—standard deviation).

**Table 2:** Digital Vs. In-person programmes.

	Digital (31)	In-Person (26)	*p* Values
Age (y, mean (sd))	67.4 (8.9)	65 (10.1)	0.37
Sex (male)	26 (84%)	17 (65%)	0.06
Stage	4-1 3-24 2-4 1-2	4-3 3-19 2-3 1-1	0.70
ASA	3–17 2–14	3–15 2–11	0.33
CCI (interquartile range)	4 (3–5)	5 (3–6)	0.08
NAC	29 (93%)	23 (88%)	0.51
Cancer (oesophagus/gastric, GOJ)	O-26 (84%) S-5 GOJ-0	O-16 (62%) S-5 GOJ-5	0.04
Procedures			
- Oesophagectomy	19 (73%)	10 (59%)	0.11
- Gastrectomy	7 (27%)	7 (41%)	
Postoperative outcomes			
- All complications	13 (50%)	11 (64%)	0.30
- Postoperative pneumonia	6 (23%)	7 (41%)	0.12
- Hospital stay (median (IQR))	10.5 (9–18)	17 (12.25–26)	0.07

(CCI—Charlson Co-Morbidity Index; NAC—neoadjuvant chemotherapy; G—gastric; GOJ—gastro-oesophageal junction; staging—as per 8th edition of UICC of malignant tumours).

## Data Availability

The data presented in this study are available on request from the corresponding author.
